# Reconstruction of a missed posterior locked shoulder fracture-dislocation with bone graft and lesser tuberosity transfer: a case report

**DOI:** 10.1186/1752-1947-2-260

**Published:** 2008-08-05

**Authors:** Byron E Chalidis, Pericles P Papadopoulos, Christos G Dimitriou

**Affiliations:** 1 Orthopaedic Department of Hippokration General Hospital, Konstantinoupoleos Street, 54642, Thessaloniki, Greece

## Abstract

**Introduction:**

Posterior shoulder fracture-dislocation is a rare emergency condition with poor prognosis when there is a delay in diagnosis and presence of associated injuries.

**Case presentation:**

We present a case of a neglected four-part fracture-dislocation of the proximal humerus in a 34-year-old Greek woman. Except from the substantially displaced and comminuted tuberosity fractures, an anterolateral defect of approximately 50% of the articular surface was apparent. Open reduction of the humeral head was followed by reconstruction of the proximal humerus with allograft impaction, transfer of lesser tuberosity to the humeral defect and anatomic fixation of the greater tuberosity and humeral neck fractures. At two and a half years postoperatively, the humeral head was revascularised and properly articulated with the glenoid fossa.

**Conclusion:**

The presented case underlines the variability of injury pattern, the potential of missed diagnosis and the need for preserving the humeral head in young patients regardless of the amount of articular surface defect and disruption of soft tissue attachments.

## Introduction

Posterior locked shoulder dislocation is an uncommon injury (2–4% of all shoulder dislocations) which may be misdiagnosed and overlooked in up to 60% of cases [1]. The spectrum of associated injuries varies from the isolated impaction fracture of the anteromedial aspect of the humeral head ("reverse Hill-Sachs lesion") to more complex fracture types of the proximal humerus (less than 1%) and shoulder girdle [1,2]. The unrecognised dislocation-fracture pattern can jeopardise the joint mobility and the vascularity of the humeral head predisposing to chronic instability, osteonecrosis and osteoarthritis [1].

We present a case of a neglected four-part posterior fracture-dislocation of the proximal humerus in a young woman. The vascularity and integrity of the humeral head were at high risk due to a large reverse Hill-Sachs lesion (50% of the articular surface) and severely displaced tuberosities fractures. Open reduction and internal fixation of the humeral neck and greater tuberosity fractures in combination with grafting and transfer of the lesser tuberosity to the humeral defect led to joint stability, viability of the humeral head and favourable functional outcome.

## Case presentation

A 34-year-old right-hand dominant Greek woman, presented at the Upper Limb Clinic of the Hospital complaining of persisting pain and stiffness in her right shoulder. The symptoms began 3 months earlier after a fall on her outstretched hand from a height of approximately 3 metres. The patient reported that the initial clinical assessment in the local emergency department and the anteroposterior radiograph of the right shoulder did not reveal any significant abnormality and a diagnosis of shoulder sprain and contusion was established. Pain medication was prescribed and a sling was applied for 10 days. After that time, the patient was re-examined and physical therapy with active and passive shoulder and upper limb exercises was commenced. As there was no improvement in pain and shoulder mobility, she was finally referred to our clinic for a second opinion and further evaluation.

**Figure 1:**
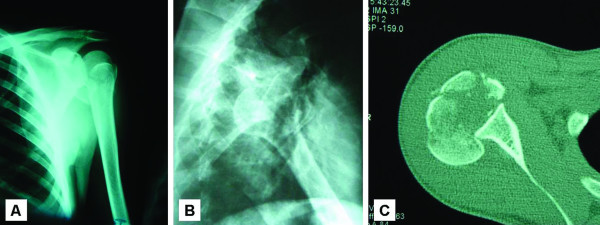
**Posterior shoulder fracture-dislocation**. A) Anteroposterior radiograph of the right shoulder showing the internally rotated humerus and the characteristic "lightbulb sign" of its proximal part. Both tuberosities have been detached from their anatomic position. B) Transthoracic lateral radiograph of the right shoulder demonstrates the posterior dislocation of the humeral head. C) Axial computed tomography (CT) scan of the right shoulder. A locked posterior fracture-dislocation is recognised. The anteromedial defect is close to 50% of the articular surface. Fracture comminution of both tuberosities and low bone density of the humeral head are also visible.

On physical examination, her shoulder looked flattened anteriorly and both acromion and coracoid processes appeared to be prominent at the anterior part of the shoulder. There was an internal rotation deformity of 30° and any effort to passively or actively move the glenohumeral joint was extremely painful. Forward elevation of 40°, no external rotation and inability to completely supinate the forearm were also identified. The patient did not have any neuromuscular deficit and her medical history was unremarkable in terms of previous injuries in the shoulder region or other medical comorbidities. The anteroposterior radiograph of the right shoulder illustrated the marked internal rotation of the proximal humerus and the typical "lightbulb sign". The greater and lesser tuberosities were fractured and displaced from each other and from the humeral head. A further undisplaced fracture line at the anatomic neck of the proximal humerus was also evident (Figure 1A). Because of the inherent patient difficulty to abduct the arm, an axillary view was not performed. The transthoracic lateral roentgenogram showed posterior extrusion of the humeral head from the glenoid fossa (Figure 1B). Furthermore, the computed tomography (CT) scan clearly delineated the locked posterior shoulder dislocation with the large anteromedial head defect (50% of the articular surface) and the comminuted fractures of both tuberosities (Figure 1C).

**Figure 2:**
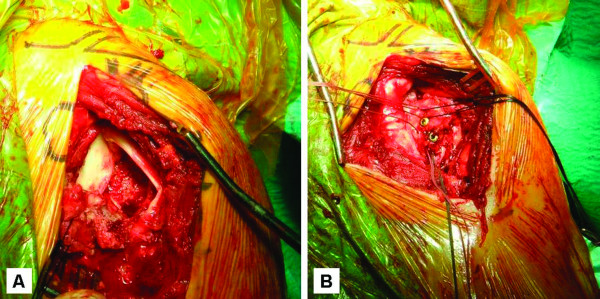
**Intraoperative photographs of the right shoulder**. A) Mobilisation of the fractured lesser tuberosity revealed the posterior dislocation of the humeral head and the "empty" glenoid fossa. B) Appearance of the right shoulder after open reduction and stabilisation of the lesser tuberosity to the anteromedial defect with two 4.0 mm titanium screws.

According to these findings, open reduction and reconstruction of the proximal humerus was considered necessary. Under general anaesthesia, the patient was placed in a beach chair position and the glenohumeral joint was assessed via a deltopectoral approach. The axillary nerve was palpated to ascertain its position but it was not mobilised. The long head of the biceps was still intact and both tuberosities were localised and circumferentially released from the newly formed granulation tissue and immature callus. As the capsule was torn and detached along with the lesser tuberosity, mobilisation of the bone fragment in a "trap-door" manner allowed easy access and visualisation of the glenohumeral joint. The humeral head was found to be dislocated posteriorly, the posterior labrum was pulled out from the glenoid and a layer of fibrous tissue covered the glenoid cavity (Figure 2A). After meticulous removal of the scar tissue, the glenoid articular cartilage looked to be in good condition and the humeral head was reduced using long Darrach retractors in combination with extra-articular pressure. However, the joint was unstable even with a few degrees of internal rotation. Using three Panalok RC (Mitek Products, Ethicon) absorbable anchors with number-2 polyester braided sutures, the posterior capsule and labrum were repaired to the posterior glenoid rim. The large reverse Hill-Sachs lesion was addressed with transfer of the fractured lesser tuberosity and its attached subscapularis muscle to the anteromedial defect according to McLaughlin's technique modified by Hawkins *et al. *[3]. Aiming to restore the sphericity of the humeral head and enhance the healing process, the bone bed of the defect was augmented with demineralised bone matrix allograft (Grafton^® ^DBM Putty, Osteotech, Eatontown, NJ) and stable fixation of the lesser tuberosity was achieved with two partially threaded 4.0 mm titanium screws (Figure 2B). The greater tuberosity and anatomic neck fractures were subsequently stabilised using three screws of the same type. Repair of the rotator interval was the last step performed and routine closure of the wound over a drain was achieved.

**Figure 3:**
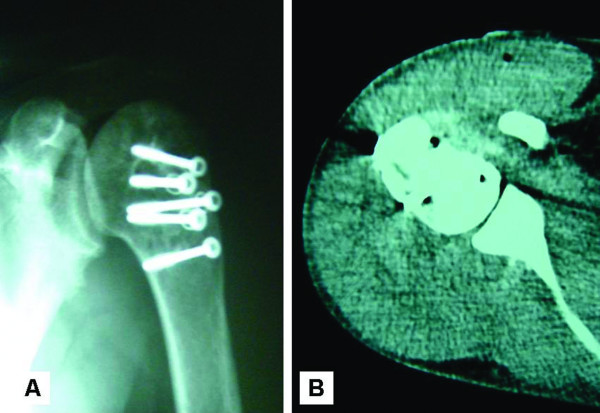
**Postoperative radiological evaluation**. A) Anteroposterior radiograph of the right shoulder at two and a half years postoperatively. The fractures have been nicely healed and the humeral head shows no signs of avascular necrosis or post-traumatic arthritis. B) At the same time, an axial computed tomography (CT) scan of the right shoulder demonstrates the well-centred humeral head over the glenoid fossa.

Postoperatively, the extremity was placed in a sling with the shoulder in neutral rotation and slight abduction. At 4 weeks, passive shoulder and pendulum exercises were initiated and the patient was advised to use the sling for another 4 weeks. At 8 weeks, a more aggressive physical therapy with active assisted range-of-motion and strengthening exercises was instituted as plane X-rays showed maintenance of joint congruency and early signs of bone healing. Despite the instructions for examination at regular intervals, the patient did not return for follow-up until two and a half years postoperatively. She reported that her shoulder was totally painless without any limitations during daily activities. She could actively elevate and abduct her arm 150° and 120°, respectively. In internal rotation, she reached the L2 vertebra and external rotation was 40°. Plane radiographs (Figure 3A) and CT scan (Figure 3B) confirmed a good clinical result and absence of devascularisation or instability of the humeral head.

## Discussion

The rarity of incidence of posterior-fracture dislocation, the potential for delay in diagnosis and the lack of evidence-based management strategies make this specific injury type challenging to treat. Recently, Robinson *et al. *[2] divided posterior-fracture dislocations into three subtypes according to the extent of fracture lines and the involvement of tuberosities. In Type I, a Neer Two-Part anatomic fracture is present without associated tuberosity fractures. In Type II, there is an additional fracture of the lesser tuberosity and in rare Type III both tuberosities are involved. The authors found the latter fracture type in 17 cases and noticed that in all of the cases, the greater and lesser tuberosities were held together giving the characteristic "shield" fragment which was first described by Edelson *et al. *[4]. Even if internal comminution exists and more fracture lines are apparent ("shattered shield" configuration), the intact periosteal sleeve averts secondary displacement. In the present case, the tuberosities were substantially displaced outlining a Neer Four-Part fracture of the proximal humerus. This finding illustrates the variability of the fracture pattern and the complexity of the underlying mechanism of injury.

Apart from the severity of injury and fracture deformity, the final prognosis is further affected by the extent of the underlying glenoid or reverse Hill-Sachs lesion [5,6]. As extensive erosion of the posterior margin of the glenoid fossa is rarely encountered even in long-standing dislocations [3], the focus is concentrated on treatment of the anteromedial defect of the humeral head. Transfer of the subscapularis or lesser tuberosity, rotational osteotomy of the humerus and allograft or autograft reconstruction have been advocated for the treatment of medium (25–40% of articular surface) or large (more than 40%) defects in cases where the articular cartilage has been impressed but not destroyed [6,7]. Hemiarthroplasty has been suggested in patients with an impression fracture involving more than 50% of the articular surface or when the humeral head is very soft and not viable [7]. However, in young patients, all efforts should be made to retain the humeral head and restore its shape, roundness and normal anatomy. Similar to our case, good results have been reported after reconstruction of defects equal to or greater than 40% of the articular surface using allograft or lesser tuberosity transfer [8,9]. Regardless of the selected treatment option, elevation of the cartilage with the adjacent bone from the impressed area and subsequent subchondral support should be carried out [1].

The transfer of lesser tuberosity instead of subscapularis alone was first introduced by Hawkins *et al. *[3]. The osteotomised or fractured bone fragment offers better filling of the defect and more secure reinsertion of the tendon [8]. Finkelstein *et al. *[10] reported that full flexion, abduction, and external rotation were achieved at 3 months in seven acutely treated shoulders with a 20% to 45% humeral head defect. The authors stated that the technique allowed earlier joint mobilisation because of the increased confidence in the immediate stability of the repaired shoulder. Checchia *et al. *[11] noted similar results but emphasised the importance of the time interval between injury and diagnosis. Specifically, posterior fracture-dislocations which were treated within 2 years of the injury had good shoulder function in comparison with neglected and misdiagnosed cases. However, Aparicio *et al. *[12] found radiographic signs of glenohumeral arthritis in six out of seven cases. The mild dislocation arthropathy was attributed to the loss of the concavity-compression effect and alteration of joint biomechanics after lesser tuberosity transfer in a non-anatomic position.

Although avascular necrosis of the humeral head is unpredictable and may occur in any posterior fracture-dislocation pattern, neglected injuries and fracture of the anatomic neck substantially increase the above incidence [13]. Accurate reduction and stable internal fixation – even if performed late – enhance the probability of successful revascularisation of the humeral head and avoid the development of avascular necrosis [14]. Head reperfusion seems to occur by the intact posteromedial vessels or alternatively by "creeping substitution" in cases with severe disruption of the arterial flow and soft tissue attachments [6]. In the presented case, the impaction of demineralised bone matrix might contribute to the viability of humeral head due to its osteoconductive and osteoinductive properties [15]. Even though it does not offer structural support, it is well suited for filling bone defects and cavities and it can be revascularised quickly. We believe that transposition of lesser tuberosity combined with allograft impaction can effectively address large humeral defects and decrease the potential of subchondral collapse or avascular necrosis.

## Conclusion

Posterior shoulder fracture-dislocation continues to be a "diagnostic trap" for the unaware physician despite the advances in imaging techniques and the continuous flow of information about the risk of missed diagnosis. In neglected injuries, open reduction of the humeral head, stable fixation of all of the associated fractures and filling of the anterolateral defect with graft and/or transfer of lesser tuberosity may lead to optimum result and good functional recovery.

## Competing interests

The authors declare that they have no competing interests.

## Authors' contributions

BEC prepared and submitted the article. PP collected and analysed the data while CGD critically revised the manuscript. Each author read and approved the final manuscript.

## Consent

Written informed consent was obtained from the patient for publication of this case report and accompanying images. A copy of the written consent is available for review by the Editor-in-Chief of this journal.
